# Supplementary material for: Spreading of P301S Aggregated Tau Investigated in Organotypic Mouse Brain Slice Cultures
Source: Biomolecules. 2022 Aug 23;12(9):1164. doi: 10.3390/biom12091164 (PMC9496515; doi:10.3390/biom12091164)
Supplement: Supplementary file 1 [file biomolecules-12-01164-s001.zip › biomolecules-1762292-supplementary.pdf]

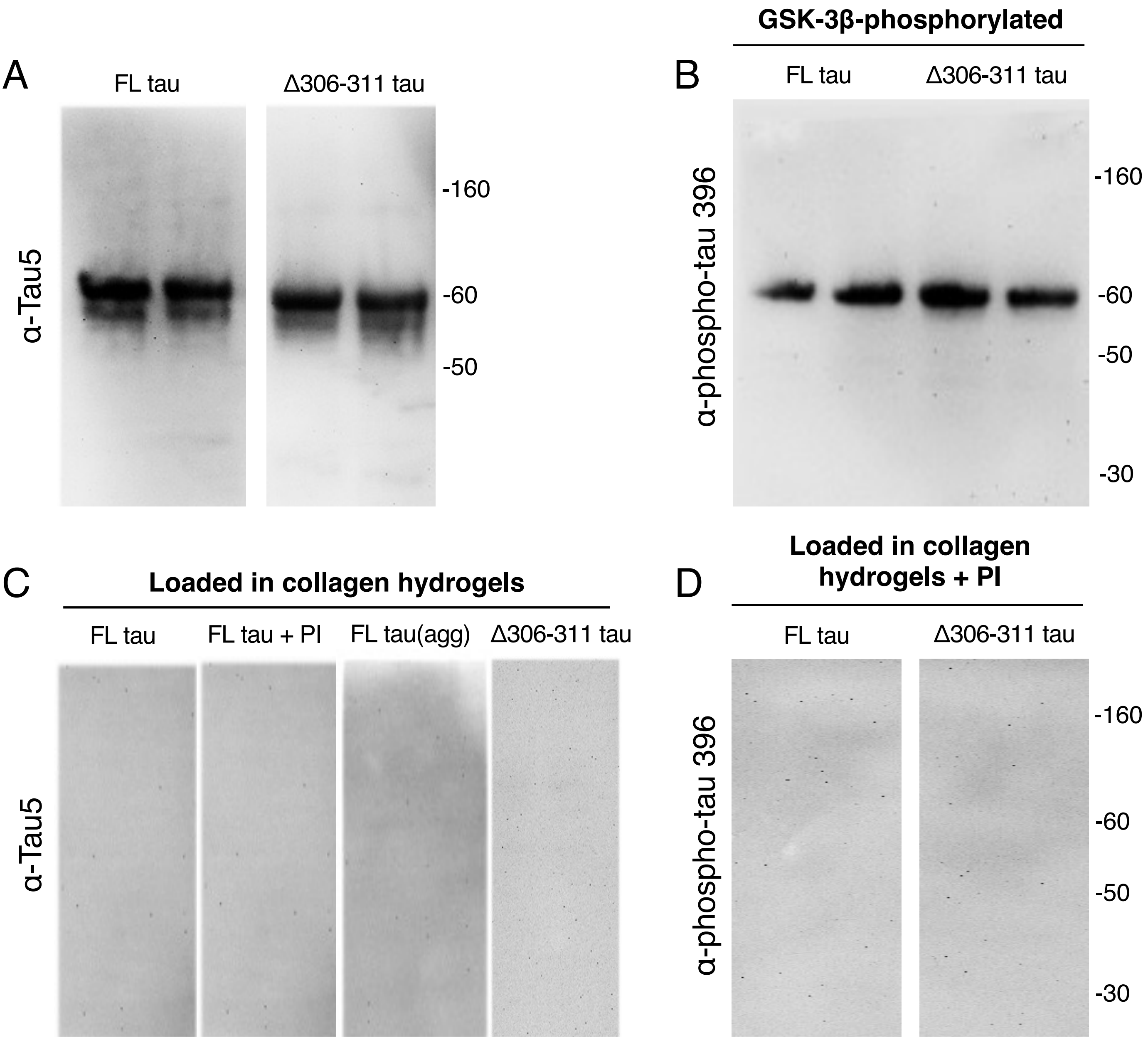

Figure S1. Characterization of FL tau and Δ306-311 tau using Western Blots.
(A) Immunostaining with the Tau5 antibody detects full-length tau (FL tau) and Δ306-311 tau as a distinct band at 60 kDa. **(B)** GSK-3β hyperphosphorylated the tau proteins, which were detectable with the phospho-tau-396 antibody. Similar to the Tau5 blot, FL tau and Δ306-311 tau are visible at the 60 kDa mark. **(C)** No signal was observed with the Tau5 antibody when FL tau, aggregated FL tau, or Δ306-311 tau were loaded into collagen hydrogels. The addition of protease inhibitors (PI) to the collagen hydrogel solution during preparation did not result in an improvement. **(D)** Loading of hyperphosphorylated FL tau or Δ306-311 tau into collagen hydrogels is also not detectable with the phospho-tau-396 antibody.

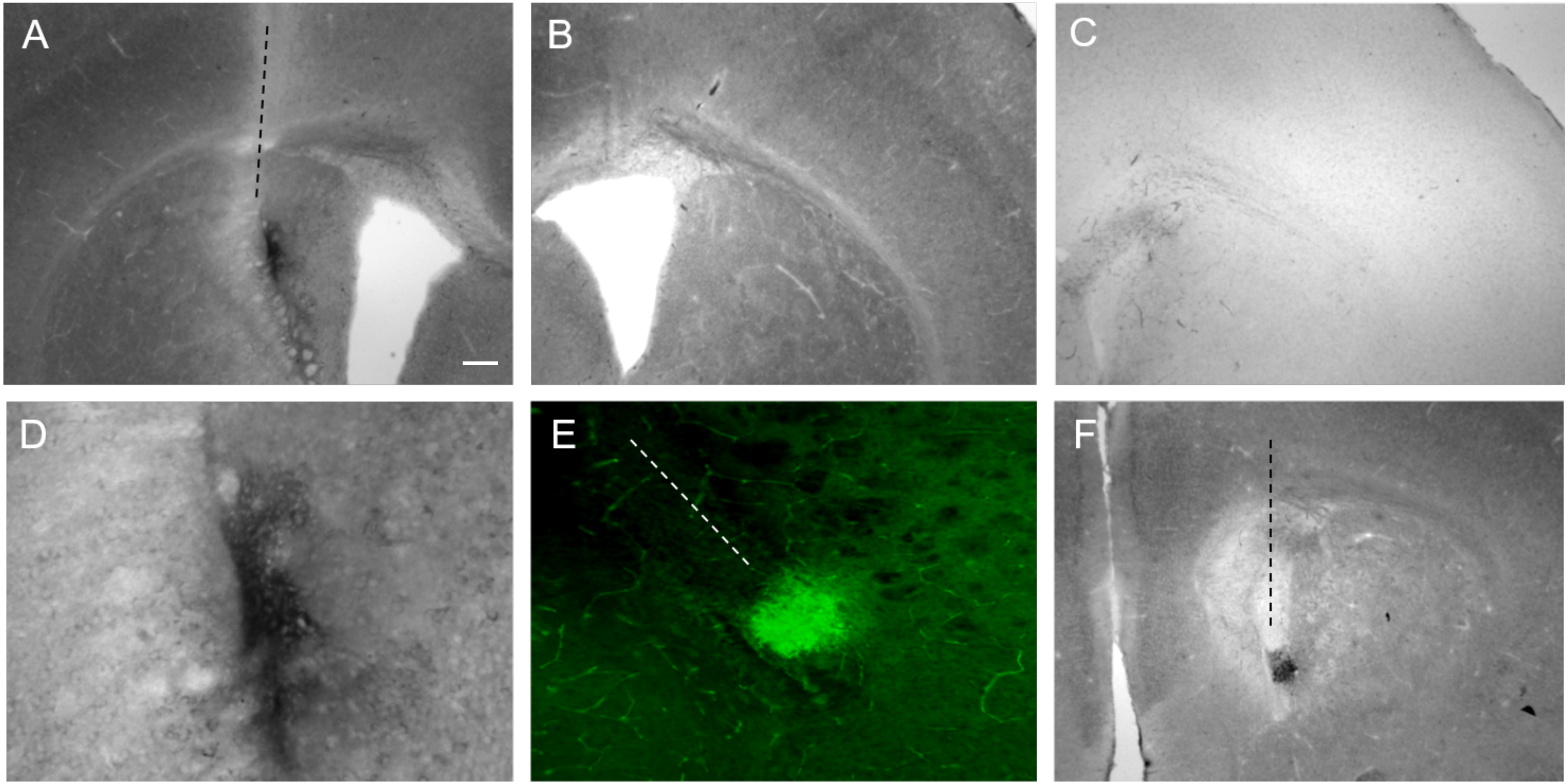

Figure S2. Immunostaining of FL tau in cryosections. A freshly dissected wild-type mouse brain was injected with 500 ng of FL tau using an injection syringe (black dotted line). Following immediate overnight post-fixation, cryosections were generated and immunostained for tau using the Tau5 antibody. **(A)** Image showing a dark staining in the approximate location of the FL tau injection. **(B)** Image of the contralateral side of the same cryosection, which displays no tau immunostaining. **(C)** As a negative control, cryosections underwent the immunostaining without the Tau5 primary antibody and no immunostaining was visible. **(D)** Tau immunostaining appears concentrated in the region of the injection in a higher magnification. **(E)** FL tau is also detectable using the fluorescence immunostaining method in the location of the tau injection (white dotted line). **(F)** Tau is detectable as a darkly stained spot compared to the slice background, even when the immunostaining protocol was performed in non-reducing or native conditions. Scale bar, 100 μm in **A-C, F**; 460 μm in **D**; 890 μm in **E**.
